# Hot Deformation Behavior of Hastelloy C276 Alloy: Microstructural Variation and Constitutive Models

**DOI:** 10.3390/ma16186192

**Published:** 2023-09-13

**Authors:** Daoguang He, Shibing Chen, Yongcheng Lin, Xintao Yan, Guan Liu

**Affiliations:** 1 School of Mechanical and Electrical Engineering, Central South University, Changsha 410083, China; 213711012@csu.edu.cn (S.C.); yxt1999charm@163.com (X.Y.); liuguan19@csu.edu.cn (G.L.); 2 State Key Laboratory of Precision Manufacturing for Extreme Service Performance, Changsha 410083, China

**Keywords:** Hastelloy C276 alloy, flow behavior, microstructural evolution, constitutive model

## Abstract

Isothermal deformation experiments of the Hastelloy C276 alloy were executed using the Gleeble-3500 hot simulator at a temperature range of 1000–1150 °C and a strain rate range of 0.01–10 s^−1^. Microstructural evolution mechanisms were analyzed via transmission electron microscope (TEM) and electron backscatter diffraction (EBSD). Results reveal that the influences of hot compression parameters on the microstructure variation features and flow behaviors of the Hastelloy C276 alloy were significant. The intense strain hardening (SH) effects caused by the accumulation of substructures were promoted when the strain rates were increased, and true stresses exhibited a notable increasing tendency. However, the apparent DRV effects caused by the annihilation of substructures and the increasingly dynamic recrystallization (DRX) behaviors occurred at high compressed temperature, inducing the reduction in true stresses. In addition, a physical-based (PB) constitutive model and a long short-term memory (LSTM) model optimized using the particle swarm optimization (PSO) algorithm were established to predict the flow behavior of Hastelloy C276 alloy. The smaller average absolute relative error and greater relation coefficient suggest that the LSTM model possesses a higher forecasting accuracy than the PB model.

## 1. Introduction

Because of the outstanding erosion resistance, antioxidant properties and high-temperature intensity, Ni-based superalloys are extensively employed in the marine industry and energy fields [1,2,3,4,5]. Hastelloy C276 alloy, a typical Ni-based superalloy, is widely used in petrochemical industries and the shielding jackets of nuclear reactors [6,7,8]. Generally, the components of Hastelloy superalloys are machined using hot deformation processes [9,10,11]. During hot deformation of Hastelloy C276 alloy, the features of flow behaviors and microstructure changes become complicated [12,13]. Therefore, investigation of the hot deformation behaviors and microstructure evolution of Hastelloy C276 alloy is crucial to precisely control the microstructures and improve the service properties [14,15].

Recently, some studies investigated the microstructure changes in alloys during hot deformation [16,17,18,19]. For example, the nucleation and coarsening features of dynamic recrystallization (DRX) were explored in numerous investigations [20,21], and multiple kinetic models were proposed for describing the DRX features of Ni-based superalloys, i.e., Hastelloy C276 alloy [22] and GH4169 alloy [23]. Moreover, the evolution mechanisms of substructures, including the dislocation clusters/loops and subgrains were explored, which impacted the DRX behaviors of Ni-based superalloys [24,25,26]. Additionally, the interaction of the precipitate phase, substructures and DRX grains in Ni-based superalloys during hot deformation [27] was revealed.

Normally, the hot flow behaviors of alloys exhibited nonlinear features and were intimately connected with the deformation parameters [28,29,30,31,32,33]. Precisely modeling of the flow behaviors was confirmed as a promising method for identifying the hot deformation features and optimizing the formation process of alloys [34,35]. Recently, multiple phenomenological models [36,37] were set up to reconstitute hot deformation behaviors of Ni-based superalloys [38]. Moreover, Chen et al. [39] developed a physically based (PB) mechanism model to accurately predict the true stresses and microstructural evolution characteristics of a nickel-based alloy in hot-working. Taking into account the combined impact of dynamic recovery (DRV) and dimples, Wen et al. [40] modeled the high-temperature tensile characteristics of an ultra-high strength steel utilizing a PB equation. Similarly, multiple PB equations were established or modified for reconstituting the hot deformation features of alloys, i.e., Hastelloy C276 alloy [41], steel [42] and Mg-9%Al-1%Zn [43]. Additionally, various artificial neural network algorithms [44,45], containing support vector regression (SVR) [46], long short-term memory (LSTM) [47] and back propagation (BP) combined with the particle swarm optimization (PSO) algorithm [48], were employed to model the hot deformation characteristics of alloys.

Previously, research was conducted to analyze microstructural changes and to model high-temperature working behaviors of Ni-based superalloys. Nevertheless, a comprehensive analysis of the evolving microstructural features and hot deformation mechanisms of the Hastelloy C276 alloy remain limited. In this present paper, the high-temperature compression features of Hastelloy C276 alloy are researched. The changes in substructures (dislocation cells, subgrain, etc.) and interaction with DRX grains are revealed. Moreover, to reproduce the flow characteristics, the PB model and the PSO-LSTM model are developed.

## 2. Materials and Methodology

The experimental material is a forged commercial Hastelloy C276 alloy and its chemical composition is shown in Table 1. The size of the experimental cylinder sample is Φ8 mm × 12 mm. Hot compression experiments with a constant strain rate are performed using the Gleeble-3500 thermomechanical simulator which is prepared by the DSI company (Nashville, TN, USA). The samples are heated to the tested temperature at a rate of 10 K/s, and held for 5 min to counterpoise the temperature gradient. Here, four compressed temperatures (1000 °C, 1050 °C, 1100 °C and 1150 °C) are selected. Then, the tested samples are hot-compressed at strain rates of 0.01 s^−1^, 0.1 s^−1^, 1 s^−1^, and 10 s^−1^, respectively. The final height reduction in the hot-compressed sample is chosen as 60%. The deformed samples are rapidly water-cooled to room temperature (*T*_0_) to maintain the microstructure after hot compression, and are analyzed using TEM and EBSD.

**Figure 1:**
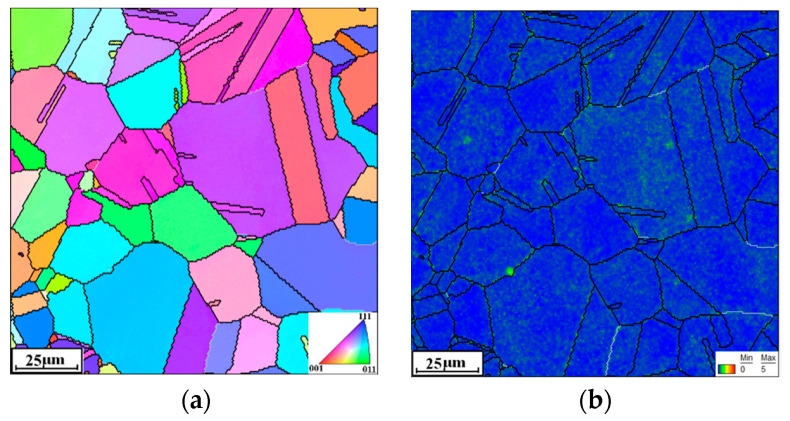
The initial microstructure of the Hastelloy C276 alloy: (**a**) inverse pole figure (IPF); (**b**) the kernel average misorientation (KAM).

For TEM and EBSD analysis, the hot-compressed specimens are sectioned along the central axis and cut into slices. Then, the slice is ground with SiC grinding papers and etched using a solution of 30 mL HClO_4_ and 270 mL CH_3_CH_2_OH. The experimental data are analyzed via the OIM software Version 6.0. The initial microstructure of the researched alloy is shown in Figure 1a, and its local misorientation is indicated in Figure 1b. The prominent grain structures are characterized as equiaxed grain and twinning grain. Moreover, few substructures can be detected.

## 3. Results and Discussion

### 3.1. High-Temperature Flow Behaviors of the Hastelloy C276 Alloy

**Figure 2:**
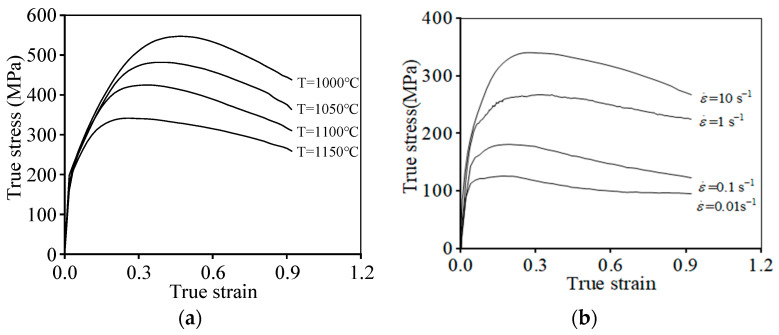
Flow stress curves of the researched alloy at (**a**) $\dot{\varepsilon }{\;=\;10\;s}^{-1}$; (**b**) T=1150 °C.

Figure 2 shows the typical true stress–true strain curves of the tested alloy at different temperatures (T) and strain rates ($\dot{\varepsilon }$). It can be distinctly seen that a rapid increase in flow stress appears at the primary strain stage of all curves. This is because of the significant work hardening effect induced by the rapid accumulation and proliferation of dislocations in this period. As the hot deformation process continues, the DRX behavior is activated, and the annihilation of substructures is accelerated. Next, the flow stress gradually reduces.

From Figure 2a, it is seen that the flow stress decreases as the T increases. The predominant cause for this is that the annihilation of dislocation loops/clusters and subgrains rotation is promoted at high T, inducing the intense DRV mechanism [49]. Moreover, the extension rate of grain boundaries is increased with increasing T, which prominently strengthens the coarsening of subgrains and the DRX grains. Additionally, the true stress exhibits an increasing tendency with the increase in $\dot{\varepsilon }$ (Figure 2b). This occurs because the progression of DRV and DRX mechanisms can be promoted at low $\dot{\varepsilon }$.

### 3.2. Microstructure Evolution

Normally, the microstructural changes in the Hastelloy C276 alloy are abruptly affected by formation parameters. Moreover, the microstructure evolution mechanism has a direct effect on the service performance of the Hastelloy C276 alloy components. Therefore, investigation of the microstructural change in the Hastelloy C276 alloy during hot working is a dire necessity.

#### 3.2.1. Effects of Compressed Temperature

**Figure 3:**
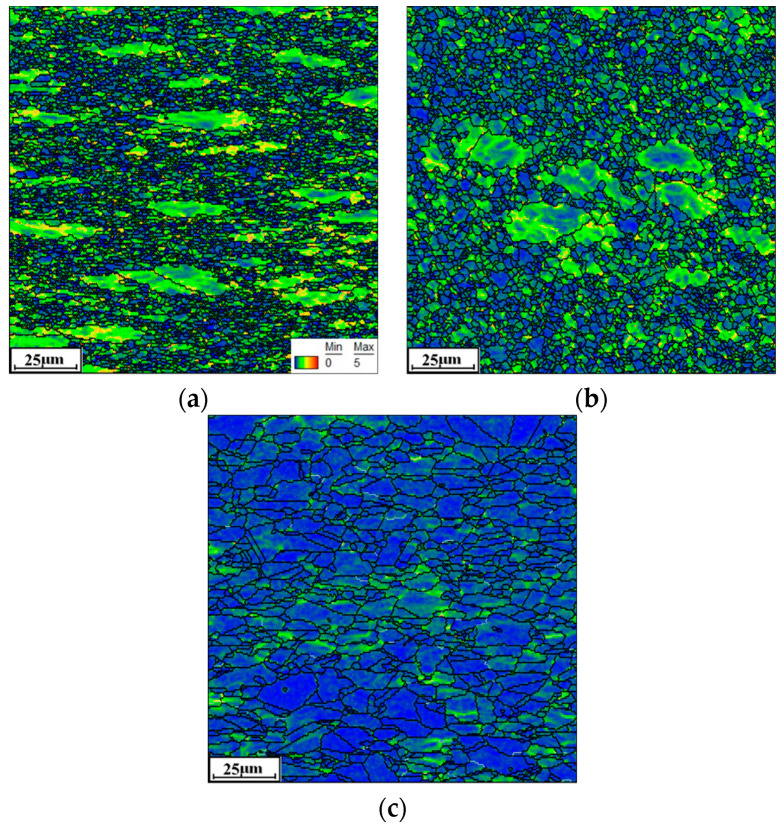
EBSD-KAM maps under different temperatures: (**a**) 1000 °C; (**b**) 1050 °C; (**c**) 1150 °C.

**Figure 4:**
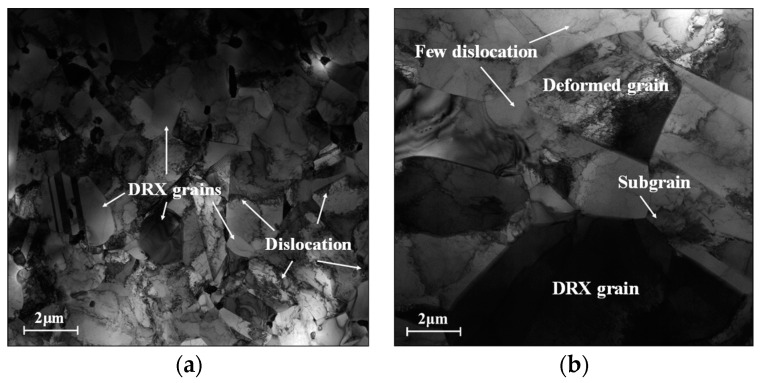
The TEM micrographs at (**a**) T = 1000 °C, $\dot{\varepsilon }$ = 0.01 s^−1^; (**b**) T = 1150 °C, and $\dot{\varepsilon }$ = 0.01 s^−1^.

Figure 3 shows the typical KAM maps of the tested Hastelloy C276 alloy at various temperatures. Accordingly, the strain rate is selected as 0.01 s^−1^. Here, the KAM angle between adjacent substructures changes from 0° to 5°, with the color changing from blue to red. For the compressed temperature (*T*) at 1000 °C, fewer areas with the blue color can be detected, suggesting the appearance of intense nucleation and interaction of substructures (Figure 3a). This is because the massive dislocation clusters, indicated by black lines, around grain boundaries or within grains are generated under complicated hot-stress conditions, as revealed in the TEM image of Figure 4a. Meanwhile, many fine DRX nucleus and subgrains are formed. With *T* increasing to 1050 °C, the areas covered in blue color experience the prominent extension, which implies the sufficient annihilation of substructures (Figure 3b). This is attributed to the abrupt increase in the DRX and DRV behaviors at high compressed temperatures, resulting in the heavy annihilation of substructures. When *T* ascends to 1150 °C, most areas are marked with blue, indicating the full development of substructures (Figure 3c). In the TEM image at 1150 °C (Figure 4b), it is discovered that the number of black lines around the grain boundaries/inside the grains tends to become significantly less, suggesting that numerous dislocations arrays/clusters are consumed through annihilation of dislocation and interaction. Concurrently, the DRX nucleus and subgrains display prominent coarsening features, which also can sweep the substructures near the grain boundaries. Hence, the elimination of substructures becomes intense with increasing *T*.

**Figure 5:**
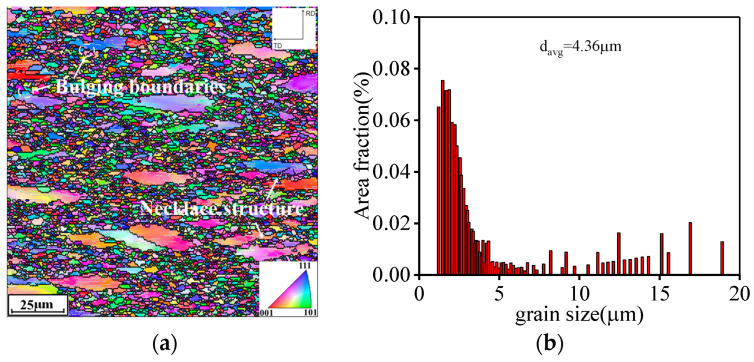

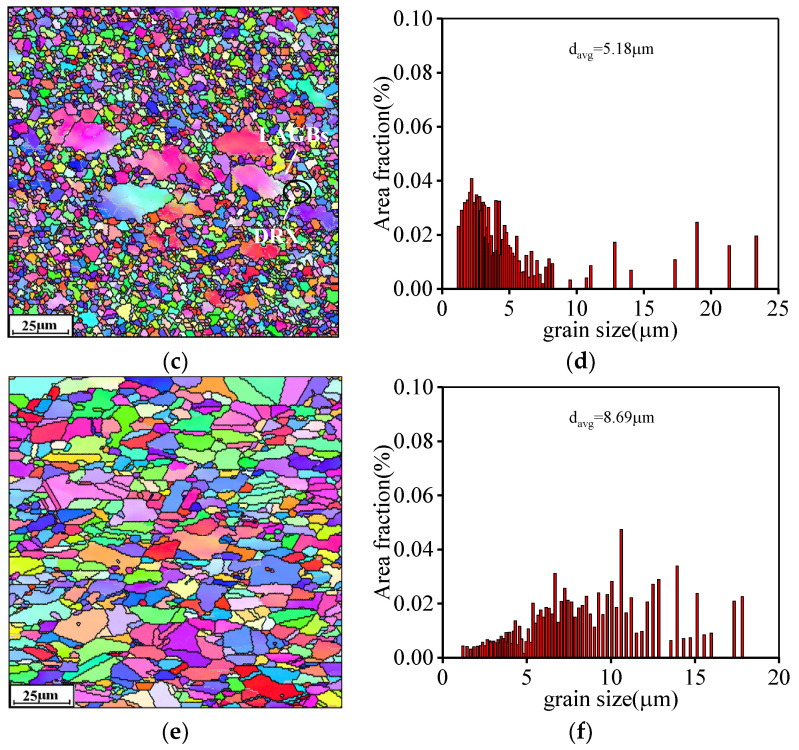
Typical EBSD-IPF maps at: (**a**,**b**) 1000 °C; (**c**,**d**) 1050 °C; (**e**,**f**) 1150 °C.

The typical evolution characteristics of grain structure over various compression temperatures are displayed in Figure 5. Accordingly, the $\dot{\varepsilon }$ is set as 0.01 s^−1^. It is seen that most of the original grains exhibit a flattened state, and the grain boundaries display bulging, which is characteristic of the researched alloys at 1000 °C (Figure 5a). Moreover, numerous fine DRX grains are generated around the bulging regions of the original grain boundaries, which forms a necklace morphology, implying the occurrence of discontinuous dynamic recrystallization (DDRX). Through statistical calculations, the mean size of grains (${\bar{d}}_{avg}$) at T of 1000 °C is computed as 4.36 µm (Figure 5b). With increasing T, almost all fine DRX grains exhibit a dominant coarsening behavior, as revealed in Figure 5c. Simultaneously, it is discovered that low-angle grains boundaries (LAGBs) form and interact, which then gradually transforms into high-angle grain boundaries (HAGBs), indicating the tendency of continuous dynamic recrystallization (CDRX). Correspondingly, the ${\bar{d}}_{avg}$ for the tested Hastelloy C276 alloy at 1050 °C reaches 5.18 µm (Figure 5d). When T gradually ascends to 1150 °C, almost all deformed original grains are replaced with DRX grains, and a dominant growth of DRX grains can be observed (Figure 5e). Concurrently, the value of ${\bar{d}}_{avg}$ ascends to 8.69 μm for the researched Hastelloy C276 alloy at 1150 °C, as shown in Figure 5f. This is because the migration rate of alloying elements and dislocations are boosted at higher T, which is beneficial for the increase in grain boundary migration ability. Additionally, the annihilation of substructures is enhanced with increasing T, thereby reducing the resistance for the mobility of DRX grain boundaries. Thus, the coarsening behaviors of grains become outstanding as T ascends.

#### 3.2.2. Effects of Strain Rate

**Figure 6:**
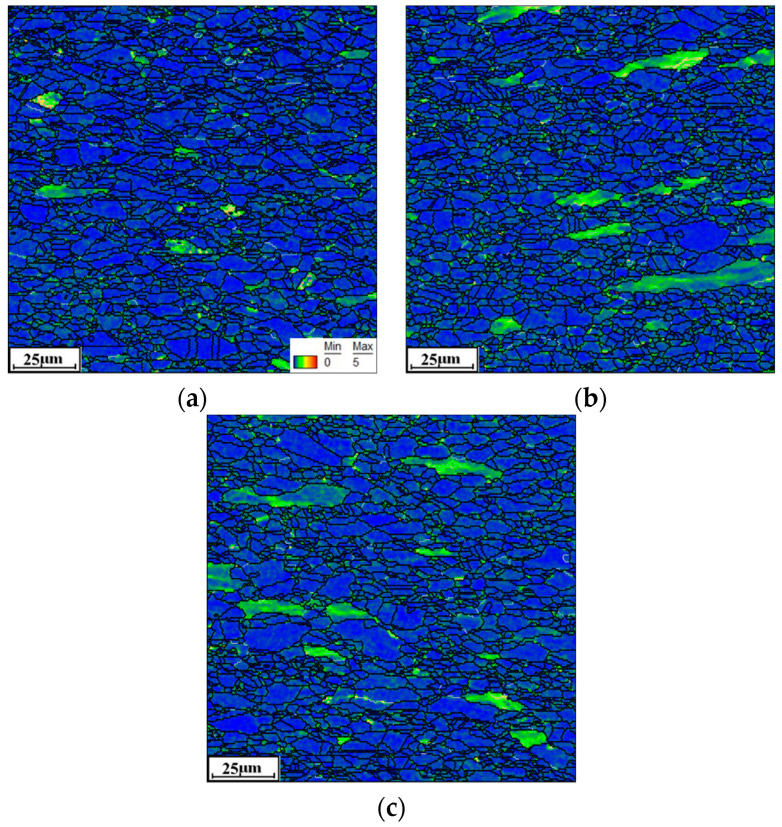
EBSD-KAM maps at the $\dot{\varepsilon }$ of (**a**) 0.1 s^−1^; (**b**) 1 s^−1^; (**c**) 10 s^−1^.

**Figure 7:**
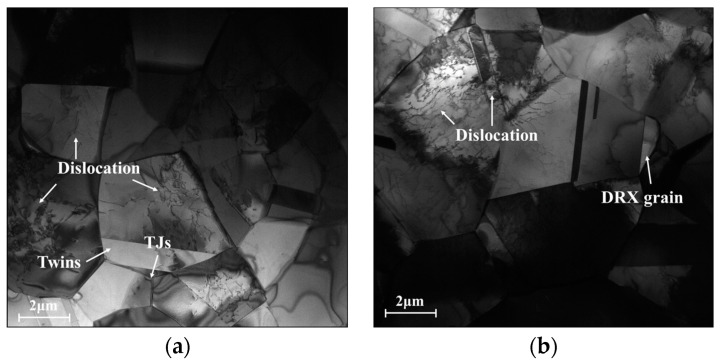
The TEM micrographs at (**a**) T = 1150 °C, $\dot{\varepsilon }$ = 1 s^−1^; (**b**) T = 1150 °C, $\dot{\varepsilon }$ = 10 s^−1^.

For T at 1150 °C, the typical KAM pictures at various $\dot{\varepsilon }$ are shown in Figure 3c and Figure 6. It is seen that the zone covered in red/yellow colors is relatively expanded at $\dot{\varepsilon }$ of 0.1 s^−1^ (Figure 6a), compared to that at 0.01 s^−1^ (Figure 3c). With the further increase in $\dot{\varepsilon }$, the zones covered in red/yellow colors are continuously extended, as shown in Figure 6b,c. This implies that the progression of LAGBs is sensibly promoted with increasing $\dot{\varepsilon }$. It is primarily caused by the formation of substructures and interaction with DRX grains. As seen in TEM pictures of Figure 4b and Figure 7, the formation of fine substructures, including the dislocation clusters/arrays and subgrains, is prominently increased at higher $\dot{\varepsilon }$. Moreover, it is also discovered that the coarsening behavior of twins and DRX grains becomes loose with increasing $\dot{\varepsilon }$. This induces the suppression of the annihilation of substructures. Additionally, the compression deformation time for the consumption of substructures through effective DRV as well as DRX mechanisms is decreased. Hence, the nucleation and interaction of LAGBs is enhanced with increasing $\dot{\varepsilon }$.

**Figure 8:**
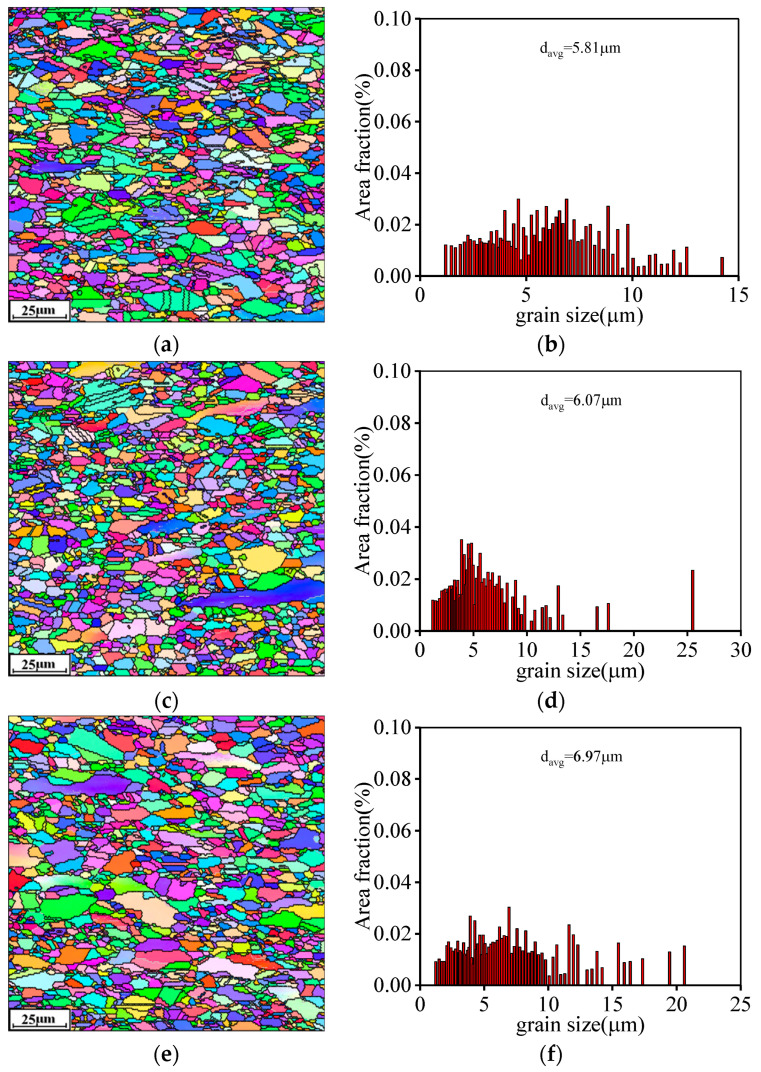
EBSD-IPF maps at: (**a**,**b**) 1150 °C/0.1 s^−1^; (**c**,**d**) 1150 °C/1 s^−1^; (**e**,**f**) 1150 °C/10 s^−1^.

The EBSD pictures highlighting the evolution of grain structure over $\dot{\varepsilon }$ are shown in Figure 8. Here, T is selected as 1150 °C. At a relatively low $\dot{\varepsilon }$ of 0.1 s^−1^ (Figure 8a), the morphology of DRX grains becomes finer compared to that at 0.01 s^−1^ (Figure 5e). Accordingly, the ${\bar{d}}_{avg}$ at the $\dot{\varepsilon }$ of 0.1 s^−1^ reaches 5.81 μm (Figure 8b). This implies that the growth behaviors of DRX grains exhibit a weakening tendency at higher $\dot{\varepsilon }$. With a substantial increase in $\dot{\varepsilon }$, the flattened original grains prominently increase, as displayed in Figure 8c,e. Simultaneously, the ${\bar{d}}_{avg}$ at the $\dot{\varepsilon }$ of 1 s^−1^ and 10 s^−1^ are statistically computed as 6.07 μm and 6.97 μm, respectively, as seen in Figure 8d,f. The primary cause for this result is that the nucleated density of substructures (as displayed in Figure 6 and Figure 7) is increased at higher $\dot{\varepsilon }$, inducing the increase in resistance to the migration of the DRX grain boundary. Additionally, the compression deformation time for the nucleation/growth of DRX grain becomes shorter with increasing $\dot{\varepsilon }$, and the remaining original grains with relatively large size are increased. Therefore, the value of ${\bar{d}}_{avg}$ is increased with the increase in $\dot{\varepsilon }$.

### 3.3. Physically Based Constitutive Model

During hot deformation, the flow stress is intimately correlated with microstructural evolution mechanism, and can be expressed as [1,39]

(1) $$\sigma =\sigma_{y}+\sigma_{i}+\sigma_{g}$$

where σ is the flow stress, and $\sigma_{y}$, $\sigma_{i}$ and $\sigma_{g}$ are the short-range stress, the stress affected by the interaction of dislocation and the stress affected by the grain size evolution, respectively.

Normally, the $\sigma_{y}$ is often computed as [50]

(2) $$\sigma_{y}=A_{y}{\left(\alpha_{y}\dot{\varepsilon }\exp\left(Q_{y}/RT\right)\right)}^{n_{y}}$$

where $A_{y}$, $\alpha_{y}$ $Q_{y}$ and $n_{y}$ represent material factors.

In hot deformation, the rapid movement and proliferation of dislocations lead to a continuous increase in internal stress. Meanwhile, the occurrence of dynamic softening mechanisms (DRV and DRX) enhances the annihilation and rearrangement of dislocations. Hence, the correlation between the dislocation density and true stress can be identified as [29]

(3) $$\sigma_{i}=M\alpha \mu b\sqrt{\rho_{i}}$$

where M is a Taylor coefficient (3.06) [39], α is an interaction constant, μ is the shear modulus (μ=85.89–0.0207T), b is the Burger vector, $\rho_{i}$ is the dislocation density and its evolution rate (${\dot{\rho }}_{i}$) is defined as [39]

(4) $${\dot{\rho }}_{i}={\dot{\rho }}_{i}^{+}-{\dot{\rho }}_{i(\mathrm{drv})}^{-}-{\dot{\rho }}_{i(\mathrm{drx})}^{-}$$

where ${\dot{\rho }}_{i}^{+}$, ${\dot{\rho }}_{i(\mathrm{drv})}^{-}$ and ${\dot{\rho }}_{i(\mathrm{drx})}^{-}$ are the evolution rate of $\rho_{i}$ connected with work hardening (WH), DRV and DRX mechanisms, respectively.

Normally, the value of ${\dot{\rho }}_{i}^{+}$ can be computed as [51]

(5) $${\dot{\rho }}_{i}^{+}=\frac{M}{b}\frac{1}{\Lambda }\dot{\varepsilon }$$

where Λ is the mean free path, and can be determined as [39]

(6) $$\Lambda =\frac{1}{s_{i}}+\frac{1}{d_{i}}$$

where $d_{i}$ is the average grain size. $s_{i}$ is the size of substructure and can be expressed as [39]

(7) $$s_{i}=f_{w}/\sqrt{\rho_{i}}$$

where $f_{w}$ illustrates the coefficient of work hardening, and can be expressed as

(8) $$f_{w}=A_{w}{\dot{\varepsilon }}^{n_{w}}\exp\left(Q_{w}/RT\right)$$

where $A_{w}$, $Q_{w}$ and $n_{w}$ are the material constants.

Normally, the $d_{i}$ can be identified as [1]

(9) $$d_{i}=X_{\mathrm{drx}}d_{\mathrm{drx}}+(1-X_{\mathrm{drx}})d_{0}$$

where $d_{0}$ and $d_{\mathrm{drx}}$ are the original grain size and the average grain size of DRX, respectively, and the $X_{\mathrm{drx}}$ indicates the DRX fraction.

For the researched Hastelloy C276 alloy in hot compression with constant strain rates, the $X_{\mathrm{drx}}$ can be defined as [22]

(10) $$\left\{ \begin{array}{l} X_{\mathrm{drx}}=1-\exp[-0.693(\frac{\varepsilon -\varepsilon_{c}}{\varepsilon_{0.5}-\varepsilon_{c}})n](\varepsilon \geq \varepsilon_{c})\\ \left\{ \begin{array}{l} \varepsilon_{c}=0.0012239Z^{0.11739}(\dot{\varepsilon }=0.1\;s^{-1}–10\;s^{-1})\\ \varepsilon_{c}=0.001543Z^{0.11366}(\dot{\varepsilon }=0.01\;s^{-1}–0.1\;s^{-1}) \end{array}\right.\\ \left\{ \begin{array}{l} \varepsilon_{0.5}=0.000815Z^{0.17507}(\dot{\varepsilon }=0.1\;s^{-1}–10\;s^{-1})\\ \varepsilon_{0.5}=0.000582Z^{0.19245}(\dot{\varepsilon }=0.01\;s^{-1}–0.1\;s^{-1}) \end{array}\right.\\ n=\left\{ \begin{array}{l} 1.512(\dot{\varepsilon }=0.1\;s^{-1}–10\;s^{-1})\\ 1.610(\dot{\varepsilon }=0.01\;s^{-1}–0.1\;s^{-1}) \end{array}\right.\\ Z=\dot{\varepsilon }\exp(\frac{467710.9}{RT}) \end{array}\right.$$

where $\varepsilon_{c}$ and $\varepsilon_{0.5}$ are the critical strain and the strain when the $X_{\mathrm{drx}}$ reaches 50%, respectively; Z is the Zener–Hollomon parameter; R expresses a gas constant (8.314 Jmol^−1^ K^−1^).

Moreover, the $d_{\mathrm{drx}}$ for the researched Hastelloy C276 alloy can be identified as [22]

(11) $$d_{\mathrm{drx}}=431.54{\dot{\varepsilon }}^{-0.10222}\exp(\frac{-46293.77}{RT})$$

Furthermore, the ${\dot{\rho }}_{i(\mathrm{drv})}^{-}$ is usually determined as [52]

(12) $${\dot{\rho }}_{i(\mathrm{drv})}^{-}=f_{v}\rho_{i}\dot{\varepsilon }$$

where $f_{v}$ is the work hardening coefficient, and can be identified as

(13) $$f_{v}=A_{v}{\dot{\varepsilon }}^{n_{v}}\exp\left(Q_{v}/RT\right)$$

where $A_{v}$, $Q_{v}$ and $n_{v}$ represent the material constants.

Meanwhile, the ${\dot{\rho }}_{i(\mathrm{drx})}^{-}$ can be defined as [39]

(14) $${\dot{\rho }}_{i(\mathrm{drx})}^{-}=\frac{f_{X}(\rho_{i}-\rho_{i0})}{\left(1-X\right)}\dot{X}\dot{\varepsilon }$$

where $\rho_{i0}$ is the initial dislocation density. $f_{x}$ is the material coefficient, and can be identified as [39],

(15) $$f_{x}=A_{x}{\dot{\varepsilon }}^{n_{x}}\exp\left(Q_{x}/RT\right)$$

where $A_{x}$, $Q_{x}$ and $n_{x}$ are the material constants.

During hot deformation, the $\sigma_{g}$ is normally defined as [52]

(16) $$\sigma_{g}=-X_{i}f_{g}d_{i}^{-0.5}$$

where $f_{g}$ is the material coefficient, and can be expressed as [39]

(17) $$f_{g}=A_{g}{\dot{\varepsilon }}^{n_{g}}\exp\left(Q_{g}/RT\right)$$

where $A_{g}$, $Q_{g}$ and $n_{g}$ are the material constants.

The above formulas are programmed into MATLAB software (Version R2021b), and the material constants in the formulas are calculated using the algorithms in the MATLAB optimization toolbox. Here, the Genetic Algorithm is employed to obtain these constants. The optimum results are listed in Table 2.

**Figure 9:**
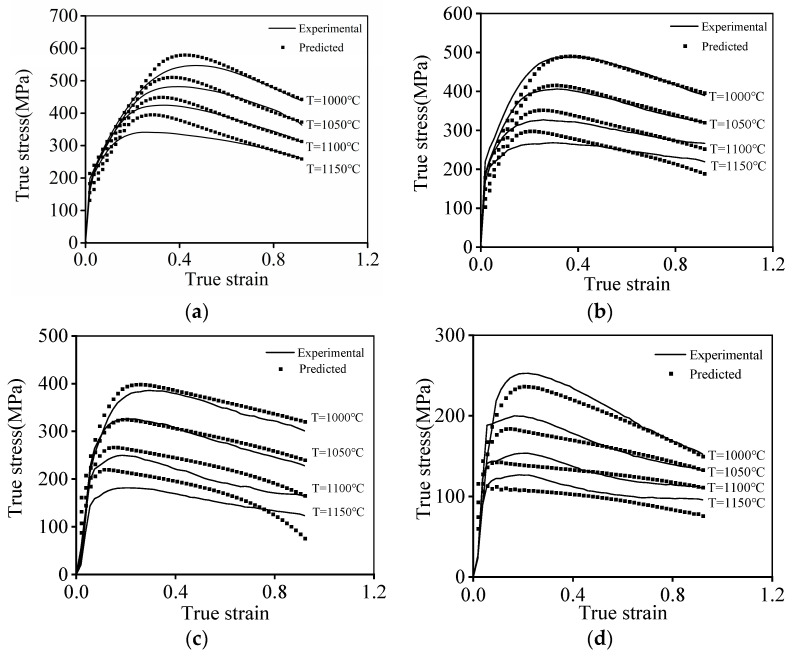
Comparison of the experimental and predicted flow stress at (**a**) $\dot{\varepsilon }$ = 10 s^−1^; (**b**) $\dot{\varepsilon }$ = 1 s^−1^; (**c**) $\dot{\varepsilon }=0.1\;s^{-1}$; (**d**) $\dot{\varepsilon }$ = 0.01 s^−1^.

Based on the above-established PB constitutive model, the forecasted hot compression curves are shown in Figure 9. Clearly, most of the tested true stresses at the $\dot{\varepsilon }$ of 10 s^−1^ and 1 s^−1^ surpassingly fit with the forecasted results of the PB model (Figure 9a,b). On the other hand, the disparity in the experimental true stresses and the ones evaluated with the PB model at a relatively low $\dot{\varepsilon }$ of 0.1 s^−1^ and 0.01 s^−1^ can be detected. This is attributed to the fact that the microstructural evolution mechanisms of the researched alloy, especially the DRX kinetic behaviors, exhibit a prominent difference as the $\dot{\varepsilon }$ is less than 0.1 s^−1^ or larger than 0.1 s^−1^. With changes in the compression conditions, the complex microstructure features also evolves, leading to the occurrence of complicated hot compression characteristics, which are hard to be ideally reconstituted using the PB model.

### 3.4. PSO-LSTM Model

**Figure 10:**
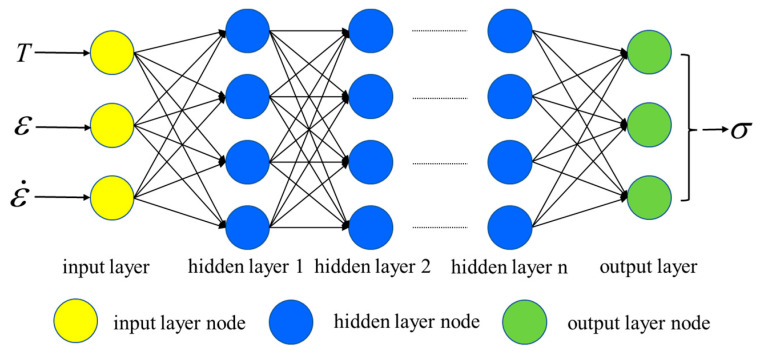
Typical structural network of an LSTM model.

Based on its excellent ability to model nonlinear data, the LSTM is adopted to reveal the hot compression behaviors of the researched Hastelloy C276 alloy. The typical structure of the LSTM model is shown in Figure 10. Here, the input layers are chosen as the T, ε and $\dot{\varepsilon }$. Correspondingly, the output layer is defined as σ. Owing to the intricacy of the stress–strain data, the value of hidden layer is set as 2 [47].

**Figure 11:**
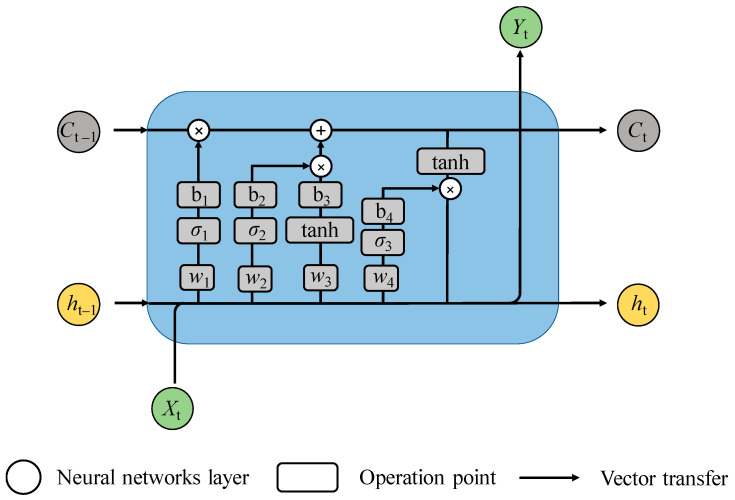
The data transmission process of the LSTM unit.

Normally, the LSTM model searches for a global optimal solution through gradient descent. The typical data transmission process of the LSTM unit is depicted in Figure 11. The forgetting gate, inputting gate and outputting gate are used to protect and control the cell state, so as to achieve the purpose of learning or forgetting information from time series data. The input data will be fed into the outputting gate, the forgetting gate, the inputting gate and the candidate gate, respectively. Then, the four gates will output different data for subsequent computation; the detailed procedures can be interpreted as follows.

Firstly, the forgetting gate ($f_{t}$) cleans up the useless input data $x_{t}$ from the last cell status, which can be expressed as [53]

(18) $$f_{t}=\sigma_{1}\left(W_{1}\cdot [h_{t-1},x_{t}]+b_{1}\right)$$

where $\sigma_{1}$ is the activation function; $\left[h_{t-1},x_{t}\right]$ is the inputting data; $W_{1}$ and $b_{1}$ represent the weight and bias of the forgetting gate, respectively.

Then, the storage status of information for the individual cell is determined. Here, the sigmoid function ($\sigma_{2}$) of the inputting gate ($i_{t}$), as the activation function, decides which kind of message will be delivered to the hyperbolic tangent activation function (tanh). The formulas of $i_{t}$ and ${\tilde{C}}_{t}$ can be determined as [47]

(19) $$i_{t}=\sigma_{2}\left(W_{2}\cdot [h_{t-1},x_{t}]+b_{2}\right)$$

(20) $${\tilde{C}}_{t}=\tanh\left(W_{3}\cdot [h_{t-1},x_{t}]+b_{3}\right)$$

where ${\tilde{C}}_{t}$ represents the unit state; $W_{2}$ and $b_{2}$ represent the weight and bias of the inputting gate, respectively. $W_{3}$ and $b_{3}$ represent the weight and bias of the cell status, respectively.

Next, the inputting gate is combined with the forgetting gate, and the cell status is refreshed from $C_{t-1}$ to $C_{t}$. The refreshed equation of $C_{t}$ can be expresses as [47]

(21) $$C_{t}=f_{t}\cdot C_{t-1}+i_{t}\cdot {\tilde{C}}_{t}$$

Therefore, the data counted in the outputting gate $o_{t}$ can be renovated and calculated using [47]

(22) $$o_{t}=\sigma_{3}\left(W_{4}\cdot [h_{t-1},x_{t}]+b_{4}\right)$$

where $\sigma_{3}$ represents the activation function of outputting gate; $W_{4}$ and $b_{4}$ represent the weight and bias of the outputting gate, respectively.

Eventually, with the introduction of the activation function (tanh), the next hidden status ($h_{t}$) can be expressed as [47]

(23) $$h_{t}=o_{t}\cdot \tanh\left(c_{t}\right)$$

Normally, the LSTM model can effectively overcome the defects of vanishing gradients and exploding gradient. However, several problems including the susceptible to fall into local minima and the relatively limited convergence rate significantly affect the applicability of the LSTM model. In order to improve convergence speed and forecasting accuracy of the LSTM model, the coupling of the PSO algorithm with the LSTM model (PSO-LSTM) is proposed to reconstruct the hot compression behaviors of the researched alloy. Commonly, the PSO algorithm is characterized as an evolutionary computing technology based on swarm intelligence. The individual positions of a PSO algorithm can be updated by tracking the individual extreme value (pbest) and the group extreme value (gbest). The velocity and position equation of particle can be, respectively, expressed as [53]

(24) $$v_{i}=W\times v_{i}+c_{1}\times rand()\times \left(pbest_{i}-x_{i}\right)+c_{2}\times rand()\times \left(gbest_{i}-x_{i}\right)$$

(25) $$x_{i}=x_{i}+v_{i}$$

where $v_{i}$ is the speed of the particles, w illustrates the inertia factor, the rand() indicates a random number from 0 to 1, $x_{i}$ is the current position of the particles, and $c_{1}$ and $c_{2}$ are the learning factors.

**Figure 12:**
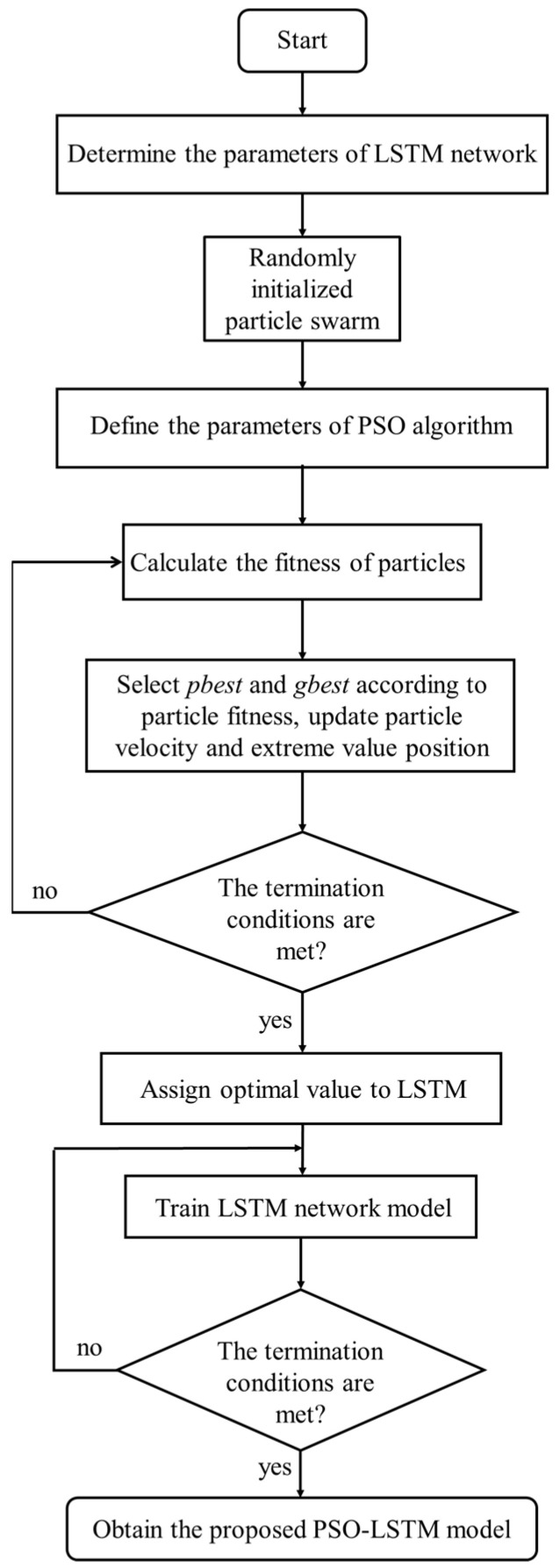
The flow chart of the PSO-optimized LSTM neural network.

Figure 12 displays the flowchart of the established PSO-LSTM model. First, the parameters of the LSTM network and the parameters (population size, evolution times, individual update range, and speed update range) of the PSO algorithm can be defined. Then, the fitness function, which is the root mean square error (RMSE) of the predicted and experimental value, can be determined. Concurrently, the pbest and gbest are updated according to the fitness. Finally, the optimal results are assigned to the LSTM model, and then the training result of the LSTM model is obtained.

Seventy-five percent of the experimental data are used for training. Correspondingly, the other twenty-five percent of the tested data are used for validating the model. According to the developed LSTM network, the length of the PSO particle matrix is set as 4. The population size is 10, and the learning factor is 2. The maximum iteration step is 100.

The total iterations of the LSTM model are set as 1500 and the min-batch size is set as 128. Concurrently, the gradient threshold is set as 1. The training method adopts Adaptive Moment Estimation (Adam). In order to ensure smooth training of the model, all experimental data are normalized as

(26) $$X_{i}=\frac{X-X_{\min}}{X_{\max}-X_{\min}}$$

where X is the input value. $X_{\max}$ and $X_{\min}$ are the maximum and minimum value, respectively.

**Figure 13:**
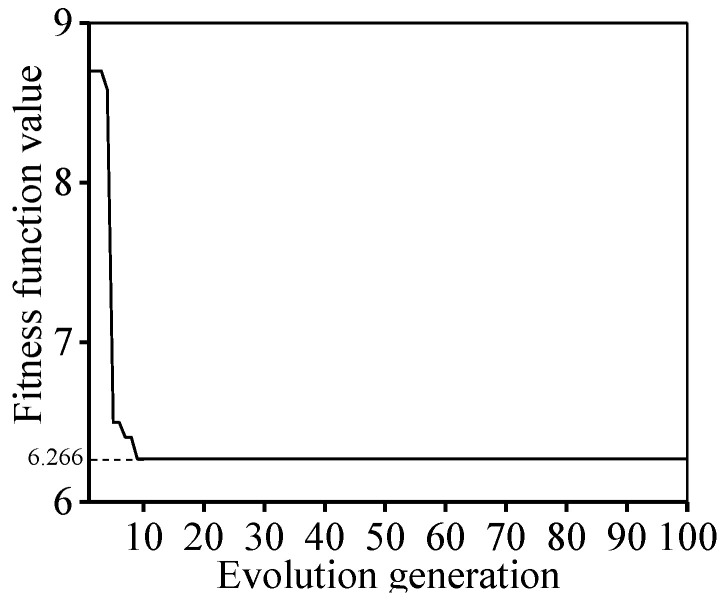
The training process of the PSO-LSTM algorithm.

After inputting the data, the PSO-LSTM model performs iterative calculations to achieve its own convergence. Usually, the fitness function is utilized to assess the convergence, as shown in Figure 13. A better convergence occurs as the fitness value is small. When the PSO algorithm iterates to the 10th generation, the fitness value of the best particle reaches the lowest point (6.266). Therefore, the optimal LSTM parameters are obtained from the PSO algorithm at the 10th generation. Here, the hidden layer 1 node is 150 and the hidden layer 2 node is 100. Accordingly, the fully connected layer node is 162, and the learning rate is 0.048. The predicted value of flow stress can be obtained by integrating the parameters into the LSTM model.

**Figure 14:**
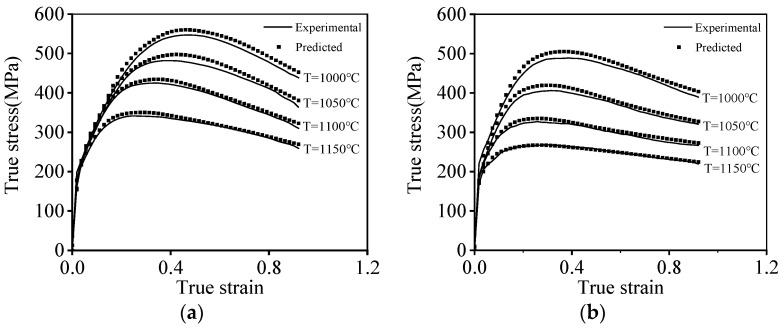

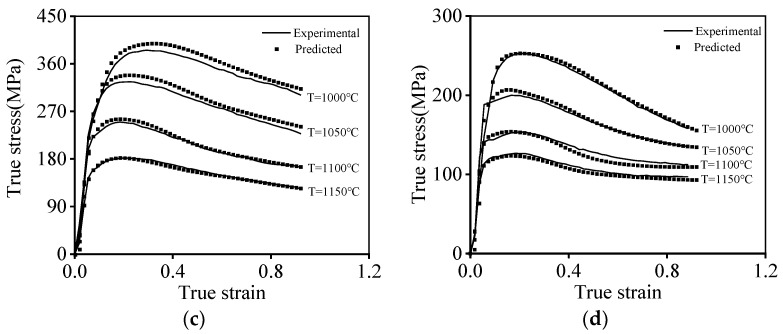
Comparison of the experimental true stress and predicted results of the PSO-LSTM model at (**a**) $\dot{\varepsilon }$ = 10 s^−1^; (**b**) $\dot{\varepsilon }$ = 1 s^−1^; (**c**) $\dot{\varepsilon }=0.1\;s^{-1}$; (**d**) $\dot{\varepsilon }$ = 0.01 s^−1^.

The forecasting capacity of the established PSO-LSTM model is shown in Figure 14. Through contrastive analysis, it is seen that the experimental true and predicted stresses generated by the PSO-LSTM model exhibit the outstanding consistency. It implies that the PSO-LSTM model can be employed to achieve accurate reconstitution of the hot compression features of the tested alloy.

### 3.5. Verification

The correlation coefficients (R) and average absolute relative errors (AARE) are often used to assess the fit of tested and forecasted data. Accordingly, the values of R and AARE are evaluated as

(27) $$R=\frac{\sum_{i=1}^{N}\left(E_{i}-\bar{E})(P_{i}-\bar{P}\right)}{\sqrt{\sum_{i=1}^{N}{\left(E_{i}-\bar{E}\right)}^{2}{\left(P_{i}-\bar{P}\right)}^{2}}}$$

(28) $$AARE=\frac{1}{N}\sum_{i=1}^{N}\left|\frac{E_{i}-P_{i}}{E_{i}}\right|$$

where $E_{i}$ and $P_{i}$ are the experimental and predicted data, respectively. $\bar{E}$ and $\bar{P}$ are the mean value of the experimental data and the predicted data, respectively. N is the volume of total data.

**Figure 15:**
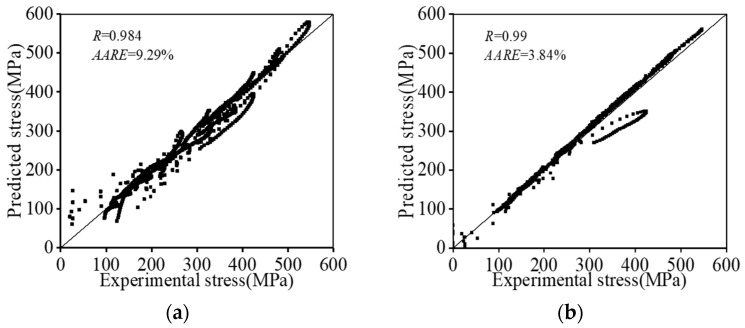
The relationship between the experimental stresses and predicted value by: (**a**) the PB constitutive model; (**b**) the PSO-LSTM model. (The black dots represent the forecasted data and the straight lines represent the standard lines).

The values of R and AARE are shown in Figure 15. It is observed that the values of R for the proposed PB model and PSO-LSTM model are computed as 0.984 and 0.99, respectively. This implies that a good correlation between the tested stresses and forecasted results via the two models can be achieved (Figure 15a,b). Additionally, the value of AARE for the PB model is computed as 9.29%, which is relatively larger than that of the PSO-LSTM model (3.84%). It suggests that the established PSO-LSTM model has better forecasting capacity for hot compression behaviors of the tested Hastelloy C276 alloy compared with that of the PB model.

## 4. Conclusions

The microstructural evolution and high-temperature compressed features of the Hastelloy C276 alloy are investigated in this paper. Several important conclusions from our analyses can be drawn:

1. Wehn the strain rate is increased, the WH effect induced by the formation of high-density dislocation clusters/arrays and subgrains is enhanced, and consequently, the true stress increases. Nevertheless, the dislocation rearrangement, caused by the promising DRV effect and the nucleation/coarsening of DRX grains, are enhanced at higher compression temperatures, thereby inducing a prominent decrease in true stress.
2. Both the PB model and the PSO-LSTM model are proposed for reconstituting the hot compression behaviors of the tested Hastelloy C276 alloy. A reasonably larger R and a smaller AARE of the PSO-LSTM model can be acquired, which suggest its higher forecasting accuracy than the PB model.

## Figures and Tables

**Table 1:** Composition of the experimental Hastelloy C276 alloy (wt. %).

Elements	C	Si	Cr	Mo	Fe	Co	W	V	P	S	Ni
Contents	0.007	0.06	15.8	16.2	6.5	1.9	4.2	0.30	0.035	0.025	Bal.

**Table 2:** The calibrated material parameters.

Material Parameter	Value	Material Parameter	Value
*A*_w_	1.696	*A*_v_	206,710.508
*Q*_w_ (kJ/mol)	294.993	*Q*_v_ (kJ/mol)	247.962
*n*_w_	0.118	*n*_v_	−0.431
*A*_g_	0.002	*A*_X_	0.004
*Q*_g_ (kJ/mol)	336.114	*Q*_X_ (kJ/mol)	249.845
*n*_g_	0.306	*n*_X_	0.366
*A*_y_	128.233	*n*_y_	7.643
*Q*_y_ (kJ/mol)	573,636.89	*n*_y_	3.907 × 10^−22^

## Data Availability

The raw/processed data acquired in present investigation cannot be shared at this time because the data also forms part of an ongoing research.
